# Using Web-Based and Paper-Based Questionnaires for Collecting Data on Fertility Issues Among Female Childhood Cancer Survivors: Differences in Response Characteristics

**DOI:** 10.2196/jmir.1707

**Published:** 2011-09-29

**Authors:** Marleen H van den Berg, Annelies Overbeek, Helena J van der Pal, A. Birgitta Versluys, Dorine Bresters, Flora E van Leeuwen, Cornelis B Lambalk, Gertjan J.L Kaspers, Eline van Dulmen-den Broeder

**Affiliations:** ^1^VU University Medical Center AmsterdamDepartment of Pediatrics, division of Oncology-HematologyAmsterdamNetherlands; ^2^VU University Medical CenterDepartment of Obstetrics & GynaecologyAmsterdamNetherlands; ^3^Emma Children’s Hospital/ Academic Medical CenterDepartment of Pediatric Oncology and Medical OncologyAmsterdamNetherlands; ^4^University Medical Center Utrecht/Wilhelmina Children's HospitalDepartment of Pediatric Hematology/Immunology and Bone Marrow TransplantationUtrechtNetherlands; ^5^Leiden University Medical CenterWillem-Alexander Children’s HospitalLeidenNetherlands; ^6^Netherlands Cancer InstituteDepartment of EpidemiologyAmsterdamNetherlands

**Keywords:** Paper, Internet, questionnaire, response, fertility, cancer survivors

## Abstract

**Background:**

Web-based questionnaires have become increasingly popular in health research. However, reported response rates vary and response bias may be introduced.

**Objective:**

The aim of this study was to evaluate whether sending a mixed invitation (paper-based together with Web-based questionnaire) rather than a Web-only invitation (Web-based questionnaire only) results in higher response and participation rates for female childhood cancer survivors filling out a questionnaire on fertility issues. In addition, differences in type of response and characteristics of the responders and nonresponders were investigated. Moreover, factors influencing preferences for either the Web- or paper-based version of the questionnaire were examined.

**Methods:**

This study is part of a nationwide study on reproductive function, ovarian reserve, and risk of premature menopause in female childhood cancer survivors. The Web-based version of the questionnaire was available for participants through the Internet by means of a personalized user name and password. Participants were randomly selected to receive either a mixed invitation (paper-based questionnaire together with log-in details for Web-based questionnaire, n = 137) or a Web-only invitation (log-in details only, n = 140). Furthermore, the latter group could request a paper-based version of the questionnaire by filling out a form.

**Results:**

Overall response rates were comparable in both randomization groups (83% mixed invitation group vs 89% in Web-only invitation group, *P* = .20). In addition, participation rates appeared not to differ (66% or 90/137, mixed invitation group vs 59% or 83/140, Web-only invitation group, *P* =.27). However, in the mixed invitation group, significantly more respondents filled out the paper-based questionnaire compared with the Web-only invitation group (83% or 75/90 and 65% or 54/83, respectively, *P* = .01). The 44 women who filled out the Web-based version of the questionnaire had a higher educational level than the 129 women who filled out the paper-based version (*P* = .01). Furthermore, the probability of filling out the Web-based questionnaire appeared to be greater for women who were allocated to the Web-only invitation group (OR = 2.85, 95% CI 1.31 - 6.21), were older (OR = 1.08, 95% CI 1.02 - 1.15), had a higher educational level (OR high vs low = 0.06, 95% CI 0.01 - 0.52), or were students (OR employed vs student = 3.25, 95% CI 1.00 - 10.56).

**Conclusions:**

Although overall response as well as participation rates to both types of invitations were similar, adding a paper version of a questionnaire to a Web-only invitation resulted in more respondents filling out the paper-based version. In addition, women who were older, had a higher level of education, or were students, were more likely to have filled out the Web-based version of the questionnaire. Given the many advantages of Web-based over paper-based questionnaires, researchers should strongly consider using Web-based questionnaires, although possible response bias when using these types of questionnaires should be taken into account.

**Trial Registration:**

Nederlands Trial Register NTR2922; http://www.trialregister.nl/trialreg/admin/rctview.asp?TC=2922 (Archived by WebCite at http://www.webcitation.org/5zRRdMrDv)

## Introduction

The number of Internet users worldwide has doubled in the past 5 years, and it is estimated there are over 2 billion users in 2010 [1]. In the Netherlands, there were up to 15 million Internet users in 2010, representing 85.6% of the Dutch population [2]. Not surprisingly, within research settings, the Internet is increasingly being used as a tool for collecting data by means of Web-based questionnaires. The use of this type of questionnaire is less time-consuming and less costly compared with the use of paper-based questionnaires. For Web-based questionnaires, no printing and mailing costs are involved and the time spent by a researcher on data entry is minimal since the returned data are already in an electronic format. In addition, studies have reported that Web-based questionnaires have fewer response errors and fewer socially desirable responses, while no differences have been found in the accuracy of the reported information between the two types of questionnaires [3-5]. Thus, Web-based questionnaires might serve as an attractive alternative to paper-based questionnaires, especially when the target study population primarily consists of relatively young respondents [6]. However, important technical and methodological issues have been raised that should be carefully considered when using Web-based questionnaires [7,8]. An important issue is obtaining representative samples of the study population with adequate response rates to secure external validity. If response rates to a Web-based questionnaire appear to be low or seem to come from a selective group, response bias is introduced and the results might be misleading [6,9].

In the past decade, several studies have investigated response rates of Web-based versus paper-based questionnaires in many different populations and in many different settings. Response rates appear to vary widely and seem to be more dependent on the population sampled than on any other factor [10-12]. Although conflicting results have been published, recent studies have demonstrated an increase in response rates to Web-based compared with paper-based questionnaires [10,11,13,14]. Employing a mixed-mode strategy, enabling patients to fill out either a Web-based or a paper-based questionnaire, seems to enhance response rates even further [15,16]. These two types of questionnaires can be offered simultaneously or sequentially, a factor that also seems to influence response rates [17].

The current study is part of a Dutch nationwide study. This study, which was initiated in 2007, examines the effects of childhood cancer and its treatment on reproductive function, ovarian reserve, and risk of premature menopause in female childhood cancer survivors. The questionnaire used in this study, of which both a paper-based version as well as a Web-based version were available, contains questions about several fertility-related issues. So far, no studies are available comparing response rates to a Web- and paper-based version of a questionnaire on fertility issues among young adult female cancer survivors. Indeed, previous studies among female childhood cancer survivors have predominantly used paper-based questionnaires, telephone interviews, and face-to-face interviews to collect data [18]. In the US Childhood Cancer Survivor Study (CCSS) as well as in the UK British Childhood Cancer Survivor Study (BCCSS), paper-based questionnaires have been sent to large cohorts of childhood cancer survivors. These questionnaires contained questions about sociodemographic items, adverse health outcomes, use of medications, lifestyle behavior, pregnancy history, and family history. Reported response rates were 82% (CCSS) and 71% (BCCSS), respectively [19,20].

In addition, studies investigating response rates to Web-based questionnaires among survivors of childhood cancer are scarce. Thompson et al [21] used a Web-based questionnaire to investigate difficulties regarding romantic relationships in childhood cancer survivors. For this purpose, 603 survivors were sent a letter by postal mail inviting them to participate. Only 60 survivors (10%) agreed to participate and filled out the Web-based questionnaire. Low response rates were also reported by Cantrell et al [22] in their study of health-related quality of life following childhood cancer. A Web-based survey was used, which was brought to the attention of potential respondents by posting a link on six different websites intended for use by survivors of childhood cancer. Although exact response rates could not be calculated, the authors reported the response rate to be low and the time needed to collect data to be long. In another study, childhood cancer survivors were recruited for a Web-based survey on physical activity through advertisements posted on cancer survivor–related websites and newsletters [23]. Since that study also used a reactive recruitment method, no true response rates could be calculated. However, the authors stated that they realize that the generalizability of their study was limited, as the recruitment method used probably had led to a specific group of survivors responding to the study invitation.

In conclusion, it is not known what response and participation rates can be expected when inviting female childhood cancer survivors to fill out a Web-based or a paper-based questionnaire on fertility issues. More specifically, no information is available on the impact of adding a paper-based questionnaire to an invitation to fill out the Web-based questionnaire. Therefore, we aimed to evaluate whether sending a mixed invitation (paper-based together with Web-based questionnaire) rather than a Web-only invitation (Web-based questionnaire only) results in higher response and participation rates for female childhood cancer survivors filling out a questionnaire on fertility issues. Furthermore, in order to identify possible response bias, differences in type of response and characteristics of the responders and nonresponders were investigated. Moreover, factors influencing preferences for either the Web- or paper-based version of the questionnaire were examined.

## Methods

Eligible survivors for the nationwide study were selected from a cohort of patients treated for childhood cancer at one of the five Dutch pediatric oncology centers or one of the two stem cell transplant centers between 1965 and 2002. Within the collaborative Dutch Childhood Oncology Late Effects Group, an electronic database has been set up in each center that includes patient and treatment details of all patients treated for cancer before the age of 18 years. Inclusion criteria for the nationwide study and the current study were identical and were defined as: female sex, having been treated for a malignancy or central nervous system tumor before the age of 18, having survived for at least 5 years, being alive, and being at least 18 years of age at study entry. Patients were excluded if they were not able to speak or read Dutch or if they had severe sequelae related to mental health.

The nationwide study consists of three components: a questionnaire, the provision of a blood sample, and a transvaginal ultrasound measurement of the reproductive organs. The last two of these components require a hospital visit. Patients can either refuse to participate or take part in one, two, or all three components of the study. For the purpose of the current report, only the questionnaire component was taken into account.

### Questionnaire and Procedures for Distribution

The questionnaire used in the study is an adaptation of a well-tested questionnaire used by the Department of Epidemiology of the Netherlands Cancer Institute in a large-scale Dutch cohort study on long-term effects of ovarian stimulation for in vitro fertilization [24]. It addresses the following issues: sociodemographic characteristics, medical history, menstrual and reproductive history, pregnancy outcomes, menopausal symptoms and menopause, and family history of cancer and family history of subfertility or infertility.

The paper- and Web-based version of the questionnaire were identical in terms of the questions asked, their wording, and their order of presentation. In the Web-based version, however, questions not relevant to the participant were automatically skipped. The Web-based version of the questionnaire was accessible for participants through a website which was specially designed for the nationwide study.

The study population for this study consisted of 277 female childhood cancer survivors from three participating centers of the nationwide study (Emma Children’s Hospital/ Academic Medical Center Amsterdam, Leiden University Medical Center, University Medical Center Utrecht/Wilhelmina Children's Hospital). These women were randomly allocated to two groups: the mixed invitation group and the Web-only invitations group.

#### The Mixed Invitation Group

Participants in the mixed invitation group received an invitation that contained a paper-based questionnaire together with an instruction sheet for the Web-based questionnaire. This instruction sheet contained a personalized username, the name of the website, and a log-in code allowing them to log in to a secured part of the website and fill out the questionnaire.

#### The Web-only Invitation Group

Participants in the Web-only group received the above-mentioned instruction sheet containing the name of the website and the log-in details alone. However, a paper-based questionnaire could be acquired by ticking this option on the informed consent form.

For practical and logistical reasons, invitations for the nationwide study (and thus for the current study) were sent out consecutively in batches consisting of invitations to 30 to 50 women. The calculation of the target sample size was based on the expected proportions of participants in both randomization groups filling out the paper-based questionnaire. Based on a previous study by Quigley et al [25], in which similar randomization groups were used, it was estimated that 77% of participants in the mixed invitation group and 27% of the participants in the Web-only invitation group would complete and return the paper-based version of the questionnaire. With 95% power and a significance level of .05, it was estimated that a minimum of 26 participants would be required in each group [26]. However, it was decided to include all women who were invited for the nationwide study during a fixed period of time (ie, January 1, 2009, through May 31, 2010), thereby assuring that the target sample size would be met.

Randomization occurred by sorting the survivors alphabetically based on the street name of their address, after which the first half of the survivors was allocated to the mixed invitation group and the second half to the Web-only invitation group.

All eligible female survivors received a study information package by postal mail consisting of an informed consent form, a refusal form, a postage-paid reply envelope, and an instruction sheet with personalized log-in details. Depending on the allocated randomization group, a paper-based questionnaire was added to this study information package. The envelope containing the study information package was sealed and put in another envelop together with a cover letter, signed by the head of the relevant pediatric oncology department, in which the study was explained very briefly. This was done in order to give survivors the chance to return the entire study information package without having to open the envelope containing this package and without having to read the extensive study information. Thus, survivors could respond to the study information package that was sent in four different ways. These were: (1) sending back a filled-out questionnaire (either Web-based or paper-based) together with a filled-out informed consent form, (2) sending back a filled-out informed consent form only in cases where the potential participant was not willing to fill out the questionnaire but was willing to take part in other parts of the study, (3) sending back a filled-out refusal form, (4) sending back the entire study information package marked return to sender. For the purpose of this study, survivors were categorized as being responders if they chose one of the four above-mentioned response options, otherwise they were categorized as nonresponders.

Participants in both groups were assured that all information provided both by the paper-based as well as the Web-based questionnaire was confidential. Moreover, it was mentioned that data provided via the Web-based version were transmitted over a secure Internet connection and could not be viewed by unauthorized persons.

### Follow up and Reminders

If an envelope appeared undeliverable because of an incorrect or nonexistent postal address, the online telephone directory was used to find the correct address. If this proved unhelpful, vital status and current address were checked by means of the Gemeentelijke Basis Administratie (Dutch Municipal Population Register).

If the questionnaire was not returned within 3 weeks, a reminder was sent by postal mail. For participants in the mixed invitation group, this reminder consisted of a letter in which the relevance of the study was again stressed and in which the individual was asked to respond. For participants in the Web-only invitation group, a paper-based version of the questionnaire was added to this reminder letter. When, after 3 weeks, still no response was received, patients in both groups were contacted by telephone and were asked to respond.

For the purpose of the current study, response time is defined as the time (number of days) elapsed between the day the envelope with the study information package was sent and the day a response was received.

### Data Analysis

Data were analyzed using SPSS for Windows, version 15.0 (SPSS Inc, Chicago, IL, USA). Descriptive statistics were used to describe differences between (1) participants allocated to the mixed invitation group and the Web-only invitation group, (2) respondents filling out the paper-based questionnaire and the Web-based questionnaire, and (3) responders and nonresponders. Independent samples *t* tests and Pearson chi-square tests were used to test whether these differences were statistically significant. A *P* value of less than .05 was considered to be statistically significant.

Multivariable logistic regression analysis was used to predict the probability of filling out the Web-based version of the questionnaire by reporting odds ratios (ORs) and 95% confidence intervals (CIs). A prediction model was developed using a backward selection procedure with a *P* value of .10 as the criterion for exclusion of variables.

## Results

### General Response Characteristics

Included in this study were 277 women. Table 1 outlines the response characteristics of the participants allocated to the two randomization groups. Response rates were comparable in both groups (83% in mixed invitation group vs 89% in Web-only invitation group, *P* = .20). In addition, participation rates—defined as the number of women who were willing to fill out the questionnaire—did not differ significantly between the mixed invitation group and the Web-only invitation group (66% or 90/137 vs 59% or 83/140, respectively, *P* = .27). Moreover, median response time was comparable in both groups. In the mixed invitation group, significantly more respondents filled out the paper-based questionnaire compared with the Web-only invitation group (83% or 75/90 and 65% or 54/83, respectively, *P* = .01). 

**Table 1:** Response characteristics of the participants receiving the mixed invitation and the Web-only invitation (n = 277)

		Mixed Invitation Group (n=137)	Web-Only Invitation Group (n=140)	*P* Value
Number of responders, n (%)		114 (83)	124 (89)	.20
Response time in days, median (interquartile range [IQR])		32.5 (54.5)	34.5 (44.8)	.51
**Reminders sent, n (%)**				
	By mail	88 (64)	93 (66)	.70
	By telephone	29 (21)	31 (22)	.84
**Timing of response, n (%)**				
	After initial invitation	54 (47)	53 (43)	
	After 1^st^ reminder (by mail)	42 (37)	45 (36)	.56
	After 2^nd^ reminder (by telephone)	18 (16)	26 (21)	
**Type of response, n (%)**				
	Returned envelope to sender	6 (5)	10 (8)	
	Refused (sent back refusal form)	18 (16)	31 (25)	.12
	Willing to participate	90 (79)	83 (67)	
**Type of questionnaire filled out, n (%)**				
	Paper-based	75 (83)	54 (65)	.01
	Web-based	15 (17)	29 (35)	

**Figure 1:**
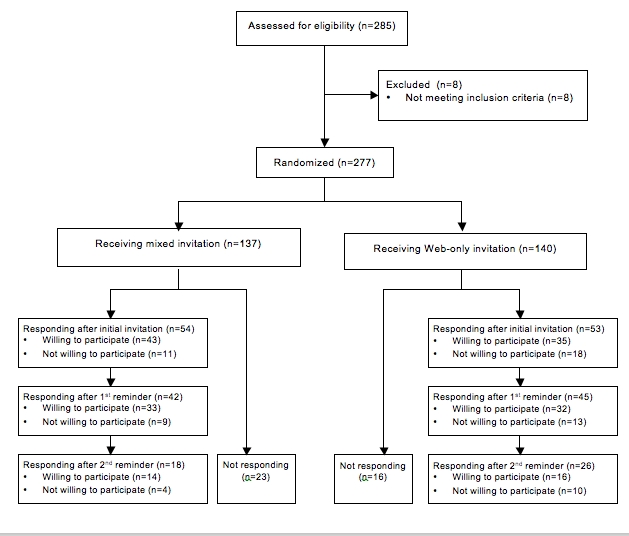
Flow diagram of participants.

Figure 1 summarizes the participant flow. Overall there were no differences between the responders in the mixed invitation group and the Web-only invitation group with respect to the number of women responding after the initial invitation, after the first reminder, and after the second reminder. However, when the results regarding the timing of the response are related to the type of questionnaire filled out by the respondents, some differences between the two groups appear. Among the group of responders who sent back either type of the questionnaire before the first postal reminder was sent, that is, the “fast responders,” the proportion of responders filling out the paper-based version of the questionnaire was significantly larger in the mixed invitation compared with the Web-only invitation group (74% or 32/43 vs 51% or 18/35, respectively, *P* = .04). This difference in type of response remained after the first postal reminder (to which a paper-based version of the questionnaire was added) was sent, but it was no longer statistically significant: 91% (30/33) in the mixed invitation group compared with 75% (24/32) in the Web-only invitation group filled out the paper-based version of the questionnaire after being reminded by postal mail (*P* = .09).

When the 238 women who responded to the study invitation by sending back the questionnaire, the informed consent form, the refusal form, or the entire study information package were compared with the 39 women who did not respond at all, it appeared that these two groups did not significantly differ regarding age, age at diagnosis, or type of diagnosis. The nonresponder group included 20 survivors (12 of 137 or 9% in the mixed invitation group and 8 of 140 or 6% in the Web-only invitation group) whose postal address could not be verified and who could not be contacted by telephone either. It was decided to consider these survivors to be nonresponders. However, they might not be “true” nonresponders since it is not known whether they indeed received the study information package and the postal reminder.

Comparing the 173 women who participated in this study with the 104 women who did not participate (ie, women indicating they refused to participate and women who did not respond) did not reveal significant differences regarding current age or age at diagnosis. However, it appeared that the proportion of women with leukemia was significantly higher in the participant group compared with the nonparticipant group (52% or 90/173 vs 38% or 39/104, respectively, *P* = .02) (data not shown).

### Characteristics of Questionnaire Respondents

Table 2 shows the characteristics of the 173 women who returned a questionnaire. It appeared that the 44 women who filled out the Web-based version of the questionnaire were more likely to have had a higher educational level than the 129 women who filled out the paper-based version (*P* = .01). No differences were found regarding age, type of diagnosis, age at diagnosis, employment status, or marital status.

**Table 2:** Characteristics of respondents who filled out the paper-based and the Web-based questionnaire (n = 173)

		Paper-Based Questionnaire (n=129)	Web-Based Questionnaire (n=44)	*P* Value
Age in years, mean ± SD (range)		29.7 ± 7.9 (18.8-52.3)	30.9 ± 8.6 (19.4-52.1)	.40
Age at diagnosis in years, mean ± SD (range)		7.4 ± 4.7 (0.4-19.5)	8.9 ± 4.6 (0.6-15.9)	.07
**Type of diagnosis, n (%)**				
	Leukemias	70 (54)	20 (45)	
	Lymphomas	17 (13)	9 (21)	
	Brain and central nervous system cancers	5 (4)	3 (7)	
	Bone tumors	10 (8)	2 (5)	.73
	Neuroblastomas	6 (5)	1 (2)	
	Germ cell tumors	3 (2)	2 (5)	
	Nephroblastomas	5 (4)	3 (7)	
	Other	13 (10)	4 (9)	
**Educational level, n (%)^a^**				
	High	30 (24)	19 (43)	
	Medium	78 (62)	24 (55)	.01
	Low	18 (14)	1 (2)	
**Employment status, n (%)**				
	Unemployed	28 (22)	4 (9)	
	Student	15 (12)	8 (18)	.12
	Employed	82 (66)	32 (73)	
**Marital status, n (%)**				
	Never married	86 (67)	29 (66)	
	Married	39 (31)	15 (34)	.56
	Divorced	3 (2)	0	

^a^ Categorized as low, up to and including lower technical and vocational training; medium, up to and including secondary technical and vocational training; and high, up to and including higher technical and vocational training and university

Among the group of respondents filling out the questionnaire, it was investigated which factors influenced the probability of filling out either the paper- or the Web-based version of the questionnaire. Table 3 shows the odds ratios and 95% confidence intervals (CI) for the variables in the final model of the logistic regression analysis. Age, educational level, employment status, and randomization group were significant factors influencing the probability of filling out the Web-based questionnaire. More specifically, the probability of filling out the Web-based questionnaire was higher for participants allocated to the Web-based invitation group for participants who were older, and for participants with a higher educational level. Finally, students appeared to have a higher probability of filling out the Web-based questionnaire compared with participants who were employed. 

**Table 3:** Factors associated with the probability of filling out the Web-based version of the questionnaire: results of logistic regressiona

		*P* value	OR	95% CI	
				Lower	Upper
Age		.01	1.08	1.02	1.15
Randomization group (reference group: mixed invitation group)		.01	2.85	1.31	6.21
**Educational level (reference group: high level)**		.04			
	Medium		0.65	0.28	1.53
	Low		0.06	0.01	0.52
**Employment status (reference group: employed)**		.03			
	Student		3.25	1.00	10.56
	Unemployed		0.35	0.10	1.29

^a^ Nagelkerke pseudo R^2^ = 0.21

## Discussion

### Statement of Principal Findings

In the present study, we examined differences in response between female childhood cancer survivors who received either a mixed invitation (paper-based questionnaire together with log-in details for Web-based questionnaire) or a Web-only invitation (log-in details only). The results show that survivors receiving the mixed invitation preferred filling out the paper-based version instead of the Web-based questionnaire as compared with the survivors receiving the Web-only invitation. Thus, when a paper-based version of the questionnaire was added to an invitation in which also the possibility of filling out the Web-based version was mentioned, the survivors were more likely to choose the paper-based option. Moreover, when the results regarding the timing of the response are taken into account this finding is endorsed since a large proportion (75%, 24/32) of females who initially received the log-in details only responded by filling out the paper-based questionnaire after they received a postal reminder (3 weeks later) to which a paper-based version of the questionnaire was added. This proportion is comparable to the proportion of females filling out the paper-based questionnaire immediately after the invitation (ie, before the postal reminder) among those who initially received the log-in details together with the paper-based version of the questionnaire (74%, 24/32).

### Comparison With Other Studies

To our knowledge, no studies are available that have compared response rates to a Web- and paper-based version of a questionnaire on reproductive and fertility issues among young adult women. However, a few studies are available that have evaluated these issues by means of a Web-based questionnaire only. In a group of female survivors of breast cancer, the response rate to this type of questionnaire was 51% [27,28] whereas in a group of women aged 17 to 21 years, this rate was 72% [29]. However, no information was provided regarding characteristics of the nonresponder group.

In our study, the overall response rates in the mixed invitation group and the Web-only invitation group did not differ. This result is in line with the results found in the study of Quigley et al [25]. In the study by Quigley et al, military personnel were requested to participate in a survey on information services. In one study group, a paper-based questionnaire was used with an added option of completing the questionnaire via the Internet. In the other study group, an Internet-based questionnaire was used with an added option of completing a paper version of the questionnaire by mail. Although response rates in both study groups were lower (42% and 37% respectively) than the response rates found in our study, differences in response rates between the two groups were not found, as was the case in our study. Furthermore, of the participants receiving the paper-based questionnaire with the Internet option, 77% chose to complete paper-based questionnaire. In our study, a similar proportion of participants in the mixed invitation group filled out the paper-based questionnaire, that is, 83%. However, Quigley et al found that of the participants receiving the Web-based questionnaire with the option of the paper-based version, 73% chose to complete the Web-based questionnaire, while in our study the proportion of women in the Web-only invitation group who filled out the Web-based questionnaire was much lower (35%).

Furthermore, the participation rates (ie, the proportion of women who filled out the questionnaire) measured in our study can be considered as being rather high (66% in the mixed invitation group and 59% in the Web-only invitation group). In other studies using Web-based questionnaires in combination with paper-based versions these rates are, in general, lower [17,25,30]. A possible explanation for the high response rates found in our study might be the salience of the study topic. It is known that potential participants are more likely to respond to both paper-based and Web-based surveys when the salience of the topic, defined as the degree to which the topic is of interest or is relevant for participants, is high [5,12,31]. Moreover, the questionnaire used in the current study was one of the three study components used in a nationwide study on fertility issues in female childhood cancer survivors, with the other two study components being the provision of a blood sample and a transvaginal ultrasound measurement of the reproductive organs. It is known that female survivors of childhood cancer are in need of information regarding their reproductive function [32,33]. Therefore, participation in this study might be appealing for a large group of the invited females, resulting in higher response rates compared with studies in which a questionnaire is the only measurement instrument used.

Our results show that the use of reminders improved the response rates substantially. After the first reminder (a letter sent by postal mail), the response almost doubled in both randomization groups. Other studies support our finding that both postal and telephone reminders are effective in increasing response rates for both Web-based surveys as well as traditional paper-based surveys [16,34,35].

In our study, the majority of the respondents preferred filling out the paper-based version of the questionnaire over filling out the Web-based version. Moreover, age, educational level, and employment status appeared to be important factors influencing the decision to fill out either version of the questionnaire. Our finding that women with a high education level as well as students tended to choose Web-based questionnaires over paper-based questionnaires is in line with previously published results [5,30,36]. However, results of the present study could not endorse other study results stating that Web-based questionnaires are more likely to attract younger respondents than paper-based questionnaires [6,36].

Another factor that may have played a role in the decision of the respondents to fill out either the paper-based or the Web-based version of the questionnaire is the length of the questionnaire used in the current study, which was rather long. The paper-based version consisted of 122 questions covering 32 pages. The Web-based questionnaire required several computer screens, with the number of questions on one screen depending on the type and length of the questions. For women filling out the Web-based version of the questionnaire, the median (IQR) time spent on filling out the questionnaire, which was automatically registered by the Web-based questionnaire tool, was 42.7 minutes (28.7 minutes to 67.8 minutes). Unfortunately, in the current study these data were not collected for the group of women filling out the paper-based version of the questionnaire. However, in the larger nationwide study, of which this study is part, a question was added to the paper-based questionnaire at a later point in time asking how much time was spent filling out the questionnaire. Median (IQR) time spent in this group (n = 145) was 30.0 minutes (30.0 minutes to 60.0 minutes) minutes. Thus, although this information was recorded among a different group of participants, it seems that filling out the paper-based version of the questionnaire took less time compared with the Web-based version. Various studies have shown the length of both Web-based and paper-based questionnaires to be negatively related to response rates [31,37-39]. However, no literature is available on differences in response rates to paper-based and Web-based questionnaires related to the length of a questionnaire. The results of our study seem to indicate that people tend to choose the paper-based version of a questionnaire when it concerns a long questionnaire. However, whether shorter surveys result in higher response rates when they are offered through the Web and longer surveys result in higher response rates when offered on paper needs further investigation.

In addition, the topic of our questionnaire can be considered to be rather personal. It is known that questionnaires containing questions of a sensitive nature result in lower response rates [31,38]. Although to our knowledge, studies investigating differences in response rates to paper-based and Web-based questionnaires taking into account the degree of sensitivity of the questions are lacking, one could assume that the sensitivity of our topic may have resulted in more respondents filling out the paper-based questionnaire especially since it is known that respondents filling out questionnaires through the Internet have doubts about their privacy and the confidentiality of their responses [10,40]. Despite the fact that data security and confidentiality were stressed in the letter accompanying our Web-based questionnaire, this might have led to more women filling out the paper-based questionnaire.

### Limitations of Current Study

An important limitation of our study is the generalizability of the results found. Our study population mainly consisted of relatively young women, and thus the results may be less representative of older age groups or mixed groups including males. Moreover, our study population represents a rather unique clinic population, that is, long-term survivors of childhood cancer. In addition, the topic of the questionnaire used cannot be considered a conventional subject. Therefore, caution should be exercised when translating the results found in the current study to other study groups or other study topics. Furthermore, the available data on the nonresponders in the present study were limited to age, age at diagnosis, and type of diagnosis. As a consequence, potential bias introduced due to nonresponse, also influencing the generalizability, could not be investigated extensively. However, in many of the studies using both paper-based as well as Web-based questionnaires, data on nonresponders are not available at all. As nonresponse to surveys seems to be increasing in recent years [41,42], future studies investigating the degree of bias as well as its consequences for the interpretation of data collected by paper-based and Web-based questionnaires are of great importance.

### Conclusions

Survivors of childhood cancer from this era represent a highly mobile group, and they may not be as available or as responsive to contact by traditional mail methods [43]. Successful recruitment of this population will require new methods of contact such as email and Web-based methods. Therefore, although our findings indicate that most survivors preferred the paper-based version over the Web-based version when offered both, we conclude that Web-based questionnaires are promising data collection tools for childhood cancer survivors. However, researchers should carefully weigh the methodological benefits and barriers of using either a paper-based or a Web-based questionnaire for this group of subjects, taking into account possible response bias.

## Abbreviations

BCCSS: British Childhood Cancer Survivor Study
CCSS: Childhood Cancer Survivor Study
CI: confidence interval
IQR: interquartile range
OR: odds ratio
SD: standard deviation
